# Nickel ions influence the transepithelial sodium transport in the trachea, intestine and skin

**DOI:** 10.1038/s41598-023-33690-2

**Published:** 2023-04-28

**Authors:** Iga Hołyńska-Iwan, Marta Sobiesiak, Wojciech Kowalczyk, Marcin Wróblewski, Anna Cwynar, Karolina Szewczyk-Golec

**Affiliations:** 1 Laboratory of Electrophysiology of Epithelial Tissue and Skin, Department of Pathobiochemistry and Clinical Chemistry, Faculty of Pharmacy, Ludwik Rydygier Collegium Medicum in Bydgoszcz, Nicolaus Copernicus University in Torun, M. Skłodowskiej-Curie 9, 85-094 Bydgoszcz, Poland; 2 Department of Inorganic and Analytical Chemistry, Faculty of Pharmacy, Ludwik Rydygier Collegium Medicum in Bydgoszcz, Nicolaus Copernicus University in Torun, Bydgoszcz, Poland; 3 Clinic of Allergology, Clinical Immunology and Internal Diseases, Dr Jan Biziel’s University Hospital No. 2, Bydgoszcz, Poland; 4 Department of Medical Biology and Biochemistry, Faculty of Medicine, Ludwik Rydygier Collegium Medicum in Bydgoszcz, Nicolaus Copernicus University in Torun, Bydgoszcz, Poland

**Keywords:** Biophysics, Cell biology, Physiology

## Abstract

Measurements of transepithelial potential and resistance in tissue and organ model systems enable the evaluation of the Ni^2+^ effect on the epithelial sodium channels, aquaporin 3, and the sodium–potassium pump in the epithelial cells. The aim of the presented study was to assess the immediate and prolonged effect of nickel ions on the transport of sodium ions in tissues exposed to direct contact with nickel, including airways, digestive tract and the skin. The influence of 0.1 mM nickel solution was performed on the trachea (n = 34), intestine (n = 44), and skin (n = 51) samples descended from 16 New Zealand albino rabbits. The electrophysiological parameters were measured in a modified Ussing chamber in stationary conditions and during a 15-s mechanical-chemical stimulation. A statistically significant decrease in the electric resistance values and the smallest range of the measured potential were observed for the Ni-treated trachea specimens. The use of nickel solution did not affect the sodium transport in the intestine epithelium. The skin fragments showed altered sodium ion transport, as demonstrated by the lower range and intensity of the measured potential. The gastrointestinal tract seems to be an organ best adapted to contact with nickel ions. In airways, nickel ions most likely enter epithelial cells and the space between them, modifying proteins and the airway surface liquid. The skin turned out to be the most sensitive tissue to the intensification of sodium ion transport through nickel ions.

## Introduction

Nickel is a naturally occurring element found in air, soil, water and living organisms. The knowledge of its biological significance increased after the discovery of the presence of Ni in the active center of the urease enzyme and was expanded by determining more nickel-containing or nickel-dependent enzymes^1^. Nickel compounds have also begun to gain interest due to their potential use in medicine and research^2–8^. Numerous nickel complexes with biological activity, including complex compounds of Ni (II) with antiepileptic drugs^4^, anticonvulsants^8^, as well as with anticancer antibiotics^6^ and vitamins^3^, have been described. Additionally, a number of nickel (II) complexes showed antibacterial^7,9^, antifungal^10,11^, anti-leishmanic^12^, anti-inflammatory^13^ and antioxidant^14,15^ properties. Attention was also paid to the anti-proliferative effect of Ni (II) complexes in relation to different cell lines^2,5,16^. Importantly, due to its properties, nickel is widely used in industry. As a result, human’s exposure to this element increases with the progressive industrialization^17^. Contact allergy to nickel is one of the most common dermatoses in industrialized countries^18,19^. Long-term exposure to Ni^2+^ and its salts can cause acute poisoning leading to conjunctivitis, inflammation of the mucous membranes of the upper airways, and the development of eczema. Chronic poisoning has been associated with an increase in the incidence of cardiovascular disease, pulmonary fibrosis and renal failure^17^.

Contact with nickel and its compounds during work increases several times the incidence of malignant neoplasms of the paranasal sinus, lung and gastrointestinal tract. Nickel contributes to the process of carcinogenesis by disrupting DNA synthesis, inducing deletions in the genetic material and inhibiting its repair processes^20^. The reduction and oxidation reactions of Ni^3+^/Ni^2+^ ions in the presence of peptides, resulting in the formation of free oxygen radicals damaging DNA, proteins and lipids, are of significant importance^17^. Ni^2+^ can also directly modify the genetic material. This is done by Ni^2+^ replacing Zn^2+^ ions in the DNA structure, which causes changes in gene expression and the formation of non-specific DNA cross-links^15,21^.

The measurement of the transepithelial electric potential (PD) during mechanical-chemical stimulation and without any stimulation, as well as the measurement of the transepithelial electric resistance (R) allow the assessment of the flow of ions in the analyzed tissue covered with epithelium^22,23^. This method can be used to assess the influence of the tested substances on the transport of sodium, chloride and other ions in the undisturbed living tissue structure fully susceptible to stimuli^23–25^. The assessment of basic sodium ion transport pathways gives an opportunity to explain primary changes occurring on the surface of cells in contact with the external environment and the substances present there^22^. Primary mechanisms associated with the change of charge on the surface of the examined tissue sample reflect the processes that occur in vivo in the organ after its direct contact with substances found in the environment^23–25^, including nickel ions. The explanation of these processes is important for the prevention of allergies, poisoning and cancerogenesis caused by nickel. Xu et al.^26,27^ proved that the initiation of hypersensitivity and allergy processes is associated with the intensification of sodium ion transport through the epithelial sodium channel (ENaC), which results in the release of low-molecular substances and the penetration of immunocompetent cells to the site with a changed electrical charge.

Taking this into account, the aim of the presented study was to assess the effect of Ni^2+^ on the transport of sodium ions in tissues normally exposed to direct contact with nickel, including respiratory tract, digestive tract and the skin. For this purpose, the influence of a 15-s and 75-min contact with Ni^2+^ on the transmembrane ion transport in fragments of the trachea, intestine and skin of experimental animals was analyzed. Due to the values of electrical resistance and permeability of substances similar to human tissues^28^, especially in the case of the skin^29^, tissue samples taken from rabbits were used in the study. The electrophysiological components of ion transport measured under stationary conditions, as well as during mechanical and mechanical-chemical stimulation, under conditions of chloride ion transport inhibition (bumetanide solution, B, 0.1 mM) for the examined tissue fragments, were assessed. It was also investigated whether the changes in the transport of sodium ions caused by nickel induce long-term changes in the measured parameters, or whether they are short-term and reversible.

## Results

Table 1 presents the results obtained for the tissue samples of the trachea, intestine and skin treated as controls, i.e. the parameters measured after the incubation of the specimens in the B solution. The transepithelial electrical resistance of the tracheal fragments incubated in the Ni solution (124 Ω/cm^2^, median) was significantly lower than in the tracheal samples incubated in the B solution (155 Ω/cm^2^, median). Additionally, a statistically significant difference between the R values measured for the trachea fragments incubated in the nickel solution and after the Ni stimulation was noticed. No statistically significant differences between the R values measured for the intestine B group (200 Ω/cm^2^, median) and for the intestine Ni group (209 Ω/cm^2^, median) were found. The resistance of the skin fragments was 904 Ω/cm^2^ (median) for the controls and 1265 Ω/cm^2^ (median) for the Ni group. There were no statistically significant differences between these results (Tables 2, 3 and 4). For R measured at the end of the experiment, i.e. after 75 min of contact with Ni^2+^ solution, the results analogous to the initial measurements were obtained for all types of tissue examined (Table 3).

**Table 1:** The electrophysiological parameters measured during 15-s mechanical-chemical stimulations by bumetanide (0.1 mM) of the trachea, intestine and skin specimens.

Parameters		B—control incubation		
		Trachea (n = 20)	Intestine (n = 22)	Skin (n = 29)
R (Ω/cm^2^)	Median	155	200	904
	Upper quartile	169	300	1158
	Lower quartile	135	137	480
PD (mV)	Median	− 1.77	− 0.4	− 0.19
	Upper quartile	− 1.19	0.09	0.14
	Lower quartile	− 2.59	− 1.87	− 1.89
PDmax (mV)	Median	− 1.5	− 0.4	0
	Upper quartile	− 0.94	0.29	0.4
	Lower quartile	− 2.32	− 3.14	− 1.74
PDmin (mV)	Median	− 1.8	− 1.1	− 0.93
	Upper quartile	− 1.43	− 0.45	0
	Lower quartile	− 2.83	− 4.18	− 2.44

The values of medians and lower and upper quartiles are given. Abbreviations: B—the specimens incubated in the bumetanide (B, 0.1 mM) solution, PD—transepithelial potential difference of tissues surface (mV) measured in stationary conditions, PDmin—minimal transepithelial potential difference measured during a 15 s stimulation of the tissue surface (mV), PDmax—maximal transepithelial potential difference measured during a 15 s stimulation of the tissue surface (mV), R—resistance (Ω/cm^2^), *p* < 0.05.

**Table 2:** Transepithelial resistance (R) of the trachea, intestine and skin measured in stationary conditions at the beginning of experiments and treated by the solution of nickel chloride (0.1 mM) and bumetanide (0.1 mM).

R (Ω/cm^2^)		Incubation (Ni)		
		Trachea (n = 14)	Intestine (n = 22)	Skin (n = 28)
Ni	Median	124	209	1265
	Upper quartile	139	257	1991
	Lower quartile	118	174	418
B	Median	124	215	1272
	Upper quartile	138	249	1979
	Lower quartile	118	178	412
Wilcoxon test (*p*)	R Ni versus R B	0.001673	0.998282	0.168065

The values of medians and lower and upper quartiles are given. Abbreviations: B—bumetanide (0.1 mM) solution, Ni—nickel chloride (0.1 mM) and bumetanide (0.1 mM) solution, R—resistance measured at the beginning of experiments (Ω/cm^2^), *p* < 0.05;

**Table 3:** Transepithelial resistance (R) of the trachea, intestine and skin measured in stationary conditions at the end of experiments and treated by the solution of nickel chloride (0.1 mM) and bumetanide (0.1 mM).

R final (Ω/cm^2^)		**Incubation (Ni)**		
		Trachea (n = 14)	Intestine (n = 22)	Skin (n = 28)
Ni	Median	145	195	1296
	Upper quartile	159	232	2193
	Lower quartile	122	186	640
B	Median	138	165	1357
	Upper quartile	247	220	1708
	Lower quartile	122	134	231
Wilcoxon test (*p*)	R Ni versus R B	0.009489	0.67432	0.20036

The values of medians and lower and upper quartiles are given. Abbreviations: B—bumetanide (0.1 mM) solution, Ni—nickel chloride (0.1 mM) and bumetanide (0.1 mM) solution, R final—resistance measured after 75 min (Ω/cm^2^), *p* < 0.05;

**Table 4:** Results of the Mann–Whitney test (*p* < 0.05) for transepithelial electric resistance measured during stationary conditions (R) for the tissue specimens.

R	*p*
Trachea	
Control versus Ni	0.000027
Intestine	
Control versus Ni	0.845538
Skin	
Control versus Ni	0.068086

Control—the specimens incubated in the bumetanide (0.1 mM) solution, Ni—the specimens incubated in the nickel chloride (0.1 mM) and bumetanide (0.1 mM) solution, R—resistance measured at the beginning of experiments (Ω/cm^2^).

The values of PD measured in stationary conditions for the tracheal fragments incubated in the B solution (Table 1) and treated with the Ni solution (Table 5) did not show statistically significant differences. However, in the case of the PD values measured after long-term incubation in the Ni solution, a statistically significant increase in the potential after the stimulation with the B solution was shown, compared to the Ni stimulation. No statistically significant differences were noted for the PD values between the nickel-treated intestine specimens (Table 5) compared to the control intestine samples (Table 1). Long-term incubation of fragments of the airways and gastrointestinal tract in the Ni solution caused no changes in the constant transport of sodium ions and did not affect the mechanisms maintaining the potential difference (Tables 5, 6 and 7). The transepithelial electric potential of the skin fragments treated with B (Table 1) was statistically significantly lower than for the fragments treated with Ni solution (Table 5). For PD measured at the end of the experiment, i.e. after 75 min of contact with Ni solution, the results analogous to the initial measurements were obtained for all types of tissue examined (Table 6).

**Table 5:** Transepithelial electric potential (PD) of the trachea, intestine and skin specimens measured in stationary conditions at the beginning of experiments and treated with nickel chloride (0.1 mM) and bumetanide (0.1 mM).

PD (mV)		Incubation (Ni)		
		Trachea (n = 14)	Intestine (n = 22)	Skin (n = 28)
Ni	Median	− 1.75	− 0.57	− 0.04
	Upper quartile	− 1.59	− 0.03	0.14
	Lower quartile	− 2.06	− 1.03	− 0.15
B	Median	− 1.67	− 0.54	0
	Upper quartile	− 1.43	− 0.02	0.15
	Lower quartile	− 2.01	− 1.29	− 0.15
Wilcoxon test (*p*)	PD Ni versus PD B	0.001859	0.118563	0.668089

The values of medians and lower and upper quartiles are given. Abbreviations: B—bumetanide (0.1 mM) solution, Ni—nickel chloride (0.1 mM) and bumetanide (0.1 mM) solution, PD—transepithelial potential difference of the tissue surface (mV) measured in stationary conditions at the beginning of experiments, *p* < 0.05.

**Table 6:** Transepithelial electric potential (PD) of the trachea, intestine and skin specimens measured in stationary conditions at the end of experiments and treated with nickel chloride (0.1 mM) and bumetanide (0.1 mM).

PD (mV)		Incubation (Ni)		
		Trachea (n = 14)	Intestine (n = 22)	Skin (n = 28)
Ni	Median	− 0.67	− 0.43	0.01
	Upper quartile	− 0.8	− 0.14	0.15
	Lower quartile	− 0.96	− 0.9	− 0.15
B	Median	− 1.12	− 0.25	0
	Upper quartile	− 0.7	− 0.37	0.1
	Lower quartile	− 1.33	− 0.64	− 0.17
Wilcoxon test (*p*)	PD Ni versus PD B	0.000187	0.072211	0.215637

The values of medians and lower and upper quartiles are given. Abbreviations: B—bumetanide (0.1 mM) solution, Ni—nickel chloride (0.1 mM) and bumetanide (0.1 mM) solution, PD—transepithelial potential difference of the tissue surface (mV) measured in stationary conditions after 75 min, *p* < 0.05.

**Table 7:** Results of the Mann–Whitney test (*p* < 0.05) for transepithelial electric potential measured during stationary conditions (PD) for the tissue specimens.

PD	*p*
Trachea	
Control versus Ni	0.800521
Control versus B	0.384578
Intestine	
Control versus Ni	0.899141
Control versus B	0.934527
Skin	
Control versus Ni	0.009406
Control versus B	0.910030

Control—specimens incubated in the bumetanide (0.1 mM) solution, Ni—specimens incubated in the nickel chloride (0.1 mM) and bumetanide (0.1 mM) solution, PD—transepithelial potential difference of the tissue surface (mV) measured in stationary condition at the beginning of experiments.

Each mechanical-chemical stimulation caused reproducible and measurable changes in the transport of ions in the airways, gastrointestinal tract and skin specimens (Table 8, Appendix: Tables A1 and A2). No statistically significant differences were observed only for the tracheal and intestine specimens during the stimulation with Ni solution compared to the control (Table 9). In the case of the gastrointestinal tract, no changes in PDmin and PDmax in relation to the B solution stimulation of the fragments previously treated with nickel were also found. The use of nickel solution apparently did not affect the sodium channels located on the surface of the intestine epithelium. In the case of the respiratory epithelium, nickel ions were possibly rinsed out with the solution of chloride ion transport inhibitor, which was confirmed by the observed smaller range of the measured potential. This observation can explain the slower absorption of sodium ions from the airway surface. The skin fragments, on the other hand, showed altered sodium ion transport, which was observed during mechanical-chemical stimulation with the Ni solution. As a result, a lower range of potential changes and a lower intensity compared to the control were shown. The use of the chloride ion transport inhibitor solution did not change the absorption of sodium ions and a single application of bumetanide did not rinse the nickel ions out from the skin surface.

**Table 8:** Minimal (PDmin) and maximal (PDmax) transepithelial electric potential measured during 15-s mechanical-chemical stimulations by nickel chloride (0.1 mM) and bumetanide (B, 0.1 mM).

		Ni incubation (Ni)					
		Trachea (n = 14)		Intestine (n = 22)		Skin (n = 28)	
Stimulation	Parameters	PDmin (mV)	PDmax (mV)	PDmin (mV)	PDmax (mV)	PDmin (mV)	PDmax (mV)
Ni	Median	− 2.04	− 1.62	− 1.01	− 0.46	− 0.18	0.15
	Upper quartile	− 1.82	− 1.37	− 1.65	0.21	− 0.34	0.49
	Lower quartile	− 2.44	− 1.91	− 0.37	− 0.98	0	0
B	Median	− 1.88	− 1.39	− 1.10	− 0.24	− 0.21	0.18
	Upper quartile	− 1.68	− 1.03	− 0.46	0.24	− 0.03	0.64
	Lower quartile	− 2.22	− 1.68	− 1.95	− 1.1	− 0.37	0

The values of medians and lower and upper quartiles are given. Abbreviations: B—bumetanide (0.1 mM) solution, Ni—nickel chloride (0.1 mM) and bumetanide (0.1 mM) solution, *p* < 0.05;

**Table 9:** Results of the Mann–Whitney test (*p* < 0.05) for minimal and maximal transepithelial electric potential (PDmin and PDmax) measured during 15-s stimulations for the tissue specimens.

	PDmin	PDmax
Trachea	*p*	*p*
Control versus Ni	0.480189	0.961473
Control versus B	0.814912	0.136217
Ni versus B	0.041294	0.014730
Intestine		
Control versus Ni	0.096085	0.366177
Control versus B	0.258904	0.281325
Ni versus B	0.324589	0.720777
Skin		
Control versus Ni	**0.031793**	**0.011235**
Control versus B	**0.048334**	**0.001578**
Ni versus B	0.905937	0.390036

Control—the specimens incubated in the bumetanide (0.1 mM) solution, Ni—the specimens incubated in the nickel chloride (0.1 mM) and bumetanide (0.1 mM) solution, *p* < 0.05;

## Discussion

The measurement of electrophysiological parameters in the Ussing chamber allows for the evaluation of full-thickness living tissue samples with the preserved ability to respond to stimuli. The use of modified Ussing chamber enabling rinsing of the tissue surface with the test solution allowed to prove the reactivity of each tested tissue sample (Tables 1, 2, 5 and 8). The mechanical-chemical stimulations induced repetitive and measurable reactions in all analyzed types of tissue and regardless of the incubation conditions used (Appendix Tables A.1, A.2, the Wilcoxon's test). The measured potential values under stationary conditions and during mechanical-chemical stimulation resulted from repetitive changes in the transport of ions, especially sodium ions. The use of bumetanide as a blocker of the transepithelial chloride ion transport allowed to induce an increased flow of sodium ions. In this way, a model of the influence of nickel ions on the sodium ion transport path in the tested airway, digestive and skin epithelia was obtained^22,23,25^.

Nickel enters the body through the inhalation, digestive tract and through the skin^19,20,30^. 75–80% of nickel oxide aerosols remain in the lungs^19^. After oral uptake of the element, about 90% is excreted in the feces, and the rest is absorbed^20^. The element administered by parenteral route is excreted through the urinary system in the amount of about 65–75% of the dose^20,31^. The most common sources of contact with nickel ions include everyday items such as coins, jewelry, buttons, locks, bathroom fixtures, as well as water and food^32^. The permeation of nickel ions bound to a variety of chemical compounds through the skin, respiratory tract and gastrointestinal tract has been proven^20,31^. It seems that even a single and short-term contact with nickel ions may result in their penetration into the body and/or the development of hypersensitivity and allergy symptoms^33^. Thus, nickel allergy can be induced by even a short-term stimulus^34^, Additionally, skin damage can cause the development of symptoms of allergic nickel dermatitis^35^. The rate of penetration of nickel ions into the body is influenced by the compactness of the barrier that ions have to overcome and by the condition of the cells that constitute the structure^18,20,36^. It has been shown that nickel can be captured by a divalent metal transporter^37^. Damage to the respiratory epithelium and the inhalation of small nickel particles can cause its faster penetration and action on the body. In the digestive tract, the rate of penetration is influenced by food filling and the presence of inflammation^31,32,36^. But in the skin, the degree of moisture and the presence of damage, including microdamage of the skin-epidermal barrier, are important^26,34,35^. It appears that the decrease in resistance may be caused by the influence of nickel ions on the adhesion and tightness of the epithelium in the airways. As nickel ions did not affect the viability and compactness of the structure of the skin and intestine specimens, these tissues seem to be adapted to the action of metal ions. Alkstrom et al.^35^ proved that only a prolonged and repeated stimulus can cause changes in the cell adhesion.

### Changes in transepithelial potential during stimulation

The influence of nickel ions on ENaC and the sodium–potassium pump in cells of epithelial cultures has been demonstrated^38,39^. Additionally, nickel ions can inhibit the action of aquaporin 3, which is located on the apical surface of the respiratory epithelium and skin cells^40^. It seems that, in the airways, nickel acts directly on cells and cell-integrating proteins, causing a change in the measured transepithelial electrical resistance (Tables 2 and 3). No change of potential measured during mechanical-chemical stimulation were determined in the case of the airway tissue samples incubated in the nickel ion solution and stimulated with this solution (PDmin and PDmax, Table 8). The use of a 15-s rinsing of the airway specimens with the Ni solution did not cause an immediate change in the transport of sodium ions. It seems that, in the proposed model, nickel ions acting on the trachea specimens did not interact with neither ENaC present on epithelial cells, nor sodium channels present on nerve endings. Nickel ions have been shown to inhibit ENaC in cell cultures from dorsal skin of the annelid *Hirudo medicinalis* and *Xenopus* oocytes^38,39^. Under the analyzed conditions, the contact of the tested fragments was insufficient to induce an inhibitory effect. It can be concluded that there was no change in both hydration and the ionic composition of the airway surface liquid (ASL). However, it has been reported that nickel ions could impair the function of the cilia and their ability to move ASL and particles that enter the respiratory epithelium^41^.

### Nickel and trachea

Despite a safe inhalation dose for contact of the respiratory tract with nickel established at the level of 0.5 mg/cm^2^/week^42^, the use of the nickel solution at the concentration of 0.1 mM (5.869 mg Ni^2+^/L) for 75 min caused a reduction in the compactness and adhesion of the trachea epithelium cells, measured as transepithelial electrical resistance (Tables 2 and 4). It could be assumed that the respiratory tract is not well adapted to prolonged contact with heavy metals^20,41^, especially in the form of an aqueous solution^40^. An observed decrease in the resistance of the fragments incubated in the Ni solution may be related to a change in the structure and/or composition of ASL-forming proteins. Moreover, an impact on the functioning of receptors on the surface of the respiratory epithelium might be associated with this effect^41^. Due to the fact that the resistance of the tracheal fragments was decreased, but remained at the level of 124 Ω/cm^2^ (median), it can be concluded that nickel ions did not affect the tightness of tight joints and cell adhesion. Increased transport of sodium ions in the trachea samples incubated in the Ni solution might be explained by the interaction of nickel ions with the sodium–potassium pump, the activity of which generates and maintains the transepithelial potential difference, and/or by the influence of Ni on ENaC present on the surface of the respiratory epithelium. However, the differences between the PDmax and PDmin values found for the tissue samples long-term treated with the Ni solution during stimulation, seem to be transient and reversible under the analyzed conditions.

### Nickel and intestine

In the digestive tract, nickel ions are transported by the divalent metal transporter 1, thus preventing the absorption of important micronutrient metals, including iron, zinc and copper ions^31,37^. In the presented study, no changes in the potential measured in the stationary conditions of the intestine fragments treated with the Ni solution were observed. There were also no changes during the mechanical-chemical stimulation, comparing the short-term and long-term reactions to nickel ions compared to the control reaction. The nickel ion solution used in the analyzed system did not affect the permeability and/or compactness of the intestine epithelium. Among the analyzed epithelial tissues, the fragments of the intestine seem to be adapted very well to contact with nickel ions. Undisturbed transport of sodium ions is extremely important for maintaining the proper level of absorption of water and nutrients, such as glucose and amino acids^31,32,36^. However, when analyzing the effect of nickel ions on the gastrointestinal tract in vivo, it is necessary to take into account food filling, the degree of its fragmentation, the presence of nutrients and the amount of available water^32,36^. The more hydrated food, rich in phosphates and phytates, the lower the absorption of nickel ions^31^. Assessing the effect of nickel ions on the physiology of the gastrointestinal tract, in addition to food filling, the iron deficiency^31,36^ and inflammatory conditions such as irritable bowel syndrome^32^ should be taken into account. Increased nickel absorption and the development of disease symptoms have been demonstrated for irritable bowel syndrome depending on exposure to nickel compounds^32,36^. It should be noted that the fragments of the intestine were collected from healthy animals and the epithelium showed no signs of inflammation or other pathological changes. Therefore, even 75 min of nickel ion contact with the intestinal epithelium caused no changes in the ion transport. However, the digested food has rather longer contact with the intestinal epithelium^31,32^, which allows many substances, including nickel ions, to act on the cells.

### Nickel and skin

Nickel ions have the ability to penetrate the skin cells arranged in individual layers^20,30,34,35^. The rate of penetration and the degree of impact on the cells is influenced by the size of the applied metal-containing molecules as well as the density of the tissue and the possible degree of its damage^30^. A nickel solution at a concentration of 2 mM was found to cause the appearance of a few min-long sodium current recorded with the patch-clamp technique in cultures of epithelial cells^38^. The use of amiloride, an ENaC inhibitor, abolished the observed effect of nickel action. The authors indicated that nickel acted by activating ENaC and the sodium–potassium pump^38^. Effect on ENaC was detected one minute after the contact, but it was transient^38^. In the presented study, the effect of nickel ions at the concentration of 0.1 mM on the transport of sodium ions in the tested skin fragments during both immediate (15 s) and long-term (75 min) contact was demonstrated. Nickel ions, penetrating between the cells, could disturb the balance, leading to the changes in the constant transport of sodium ions. Thus, the change in electronegativity might be caused by an increased transport of sodium ions from the cells to the intercellular space. In addition, nickel binding to chloride ions can change the charge to a more positive one, especially on the surface of sweat gland tubules^34^. Moreover, during the prolonged treatment of the skin fragments with nickel ions, a change in the PD measured under stationary conditions compared with the control was demonstrated (Table 7). The intensification of sodium ion transport appears to involve both prolonged opening of ENaC and the activation of the sodium–potassium pump. Increased sodium ion transport is associated with the appearance of hypersensitivity and/or allergies due to changes in hydration and osmolality of keratinocytes and their microenvironment^26,27,43^. Activation of ENaC in keratinocytes causes a strong influx of sodium ions to the cells and the simultaneous ejection of small-molecule substances in order to equalize osmolality^26,43^. Immunocompetent cells migrate towards the ejected small molecules, initiating the inflammatory process^43^. The high concentration of sodium in the intercellular space, which may occur during the loss of water in the skin due to dehydration, overheating and/or changes in blood flow through the skin, is an additional factor stimulating inflammation^27,38^. Interestingly, Hestinek et al.^33^ proved that nickel is able to bind to low-molecular structures with a negative charge, which may be consistent with the increase in the PD obtained for the nickel-treated tissue samples (Tables 5 and 6). In addition, nickel ions themselves accelerate the migration process of immunocompetent cells by initiating the ejection of chemotactic substances by keratinocytes^38^. In the proposed research model of the rabbit skin fragments, no changes were noted in the transepithelial electrical resistance (Table 4), which might prove that the applied nickel ion concentration did not change the cell activity and skin structure density, and nickel did not increase the transepithelial ion permeability.

All studies explaining the effect of nickel on exposed tissues are extremely important for the prevention of hypersensitivity and/or allergy to this metal^33–35^. In the presented study, it has been shown that contact of nickel with the skin is crucial for the penetration and action of this metal, as other types of tissue exposed to nickel (trachea, intestine) have been found to be more resistant to its effects.

## Conclusions

The results of the presented study confirm the differences in the response of the three types of tissue examined, namely the epithelium of the airways, gastrointestinal tract and skin, to exposure to nickel ions in terms of transepithelial transport of sodium ions. The gastrointestinal tract seems to be an organ best adapted to contact with nickel ions, both in the conditions of an immediate and prolonged exposure. The airways have been found to respond by changes in electrical resistance, nickel most likely penetrating the epithelial cells and the intercellular spaces, modifying proteins and the airway surface liquid. Interestingly, the skin turned out to be the most sensitive tissue to the intensification of sodium ion transport by nickel ions. Demonstrating the differences in the effect of nickel ions on the epithelial tissue that builds organs in contact with the external environment may be helpful in determining ways to preventing poisoning and the development of allergies. In addition, knowledge about the influence of nickel ions on sodium transport in the skin may support the development of effective therapies of hypersensitivity and allergy symptoms after contact with this metal. Moreover, understanding the action of nickel ions in the respiratory epithelium might help to develop methods of cleaning the airway surface from this metal and preventing the formation of neoplasms.

## Materials and methods

### Material

The experiment was performed on 34 specimens of the trachea, 44 specimens of the intestine, and 51 specimens of the skin derived from 16 New Zealand albino rabbits, collected after animal scarification. The animals were treated and scarified in accordance with the European Union law. The animals were 2–3 months old, both sexes with average weight 3.5–4.0 kg. They were held on 12/12 day/night cycle, provided with water and certified fodder for rabbits ad libitum. The tissue specimens were collected from dead animals. The animals were asphyxiated with carbon dioxide (60%, with growing concentration from 0 to 60% in 5 min) in inhaled air. In every case, the animal death was confirmed. The organs were excised and the intestine was immediately cleaned. The skin fragments from the abdomen were mechanically shaved and the subcutaneous and adipose tissue were separated with scalpel. Whole tracheas, divided transversely into three parts, were used for the examination. The gastrointestinal specimens included fragments of the large intestine, divided into 7 parts. The trachea and intestine fragments were examined directly after collection. The skin specimens, washed in the Ringer solution (RS), were frozen in 5% dimethyl sulfoxide (DMSO) in RS in -80 °C, awaiting further analysis.

### Chemicals and solutions

The following chemicals and solutions were used in the experiment:

- RS—Ringer solution: K^+^ 4.0 mM; Na^+^ 147.2 mM; Mg^2+^ 2.6 mM; Ca^2+^ 2.2 mM; Cl^-^ 160.8 mM; HEPES 10.0 mM (4-(2-hydroxyethyl)piperazine-1-ethanosulfonic acid, 238.30 g/mol; Sigma-Aldrich, USA), which was adjusted to pH 7.4 under the control of a pH-meter; basic solution with iso-osmotic properties.
- B—bumetanide solution: 0.1 mM solution of 3-butylamino-4-phenoxy-5-sulfamoylbenzoic acid; 364.42 g/mol (Sigma-Aldrich, USA), dissolved in 0.1% DMSO (dimethyl sulfoxide; 78.13 g/mol, Sigma-Aldrich, USA) and diluted in RS. Used as an inhibitor of transepithelial transport of chloride ions.
- Ni—nickel chloride solution in bumetanide—a solution of 0.1 mM NiCl × 6 H_2_O, dissolved in B.
- Mineral compounds: KCl, NaCl, CaCl_2_, MgCl_2_, NiClx6H_2_O, were purchased from Avantor Performance Materials Poland S.A., Poland.

### Experimental procedure

The measurements of electrophysiological parameters were performed in a modified Ussing chamber in stationary conditions and during a 15-s mechanical-chemical stimulation. The modification of the Ussing chamber consisted in placing the tissue sample in a horizontal position, which allowed the stimulation of the outer layer of an examined specimen with fluid from the peristaltic pump with a flow of 0.06 ml/s (1 ml/15 s). Every tissue fragment had an area of 1 cm^2^. Mechanical-chemical stimulation of the mucous side of the trachea and intestine and the outer side of the skin was performed using the solution B or Ni, respectively. The applied mechanical-chemical stimulus imitated the movement of a falling drop of liquid on the surface of the tissue sample. The tip of the stimulation nozzle was placed 6 mm above the surface of an examined tissue sample. To equalize the pressure and eliminate excess fluid, the walls of Ussing chamber were equipped with small vents (Fig. A1). The details of the method have been described elsewhere^22^.

In the case of every examined type of the tissue, the specimens, pre-treated in the RS solution, were divided into two groups and completely immersed in the solution B or Ni, respectively. Then, the samples were incubated for 30 min at room temperature according to the following scheme:

1. Control group (B, 20 samples of the trachea, 22—of the intestine, 29—of the skin): the specimens incubated in the bumetanide solution (0.1 mM) to inhibit the chloride transmembrane transport.
2. Nickel group (Ni, 14 samples of the trachea, 22—of the intestine, 28—of the skin): the specimens incubated in nickel chloride solution (0.1 mM) containing bumetanide (0.1 mM).

**Figure 1:**
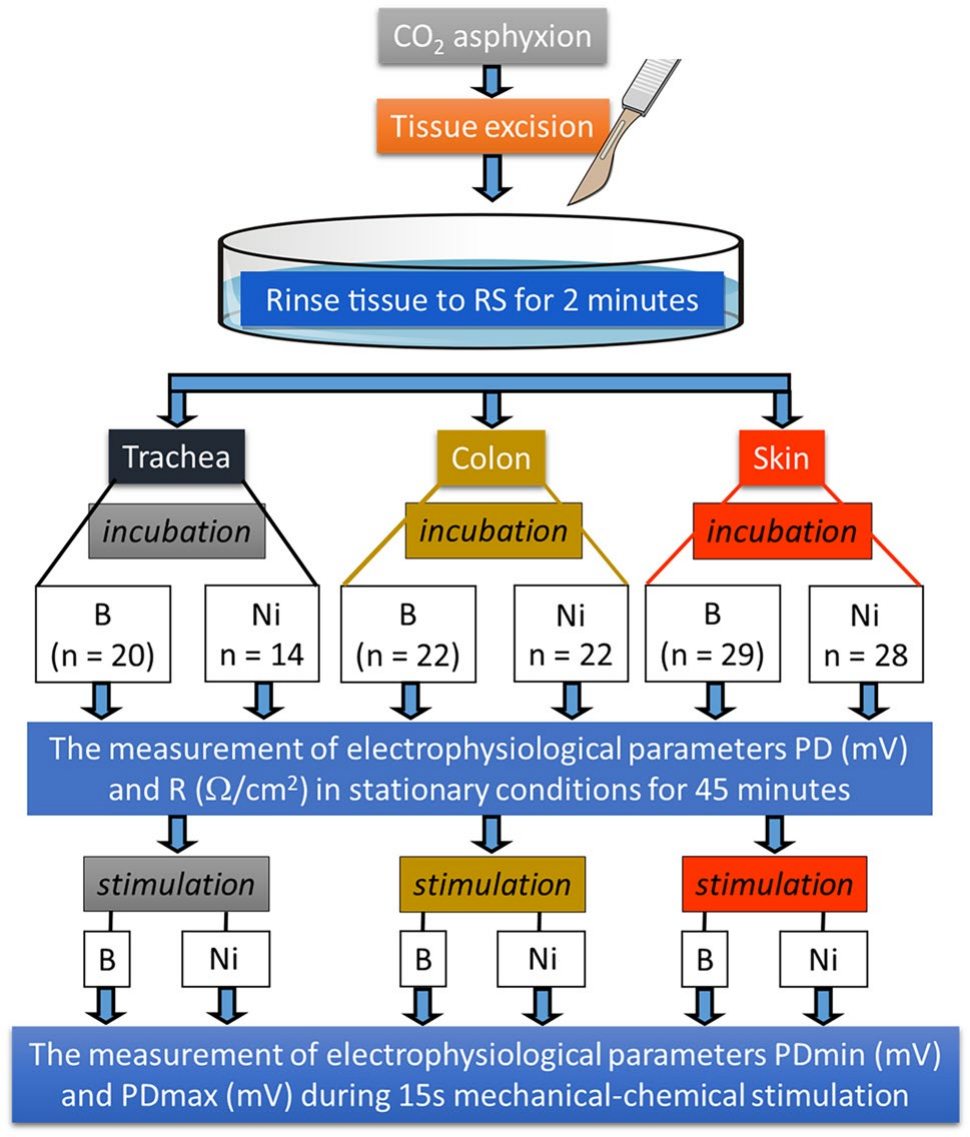
Study design. Abbreviations: RS—Ringer solution, B—control group incubated in bumetanide (0.1 mM) solution, Ni—nickel chloride (0.1 mM NiCl × 6 H_2_O) solution in B, PD—transepithelial potential difference of the tissue surface measured in stationary conditions (mV), PDmax—maximal transepithelial electrical potential measured during 15 s stimulation of the tissue surface (mV), PDmin—minimal transepithelial electrical potential measured during a 15 s stimulation of the tissue surface (mV), R—transepithelial electrical resistance (Ω/cm^2^).

After stabilization of electrophysiological parameters for all fragments, a series of stimulations was performed according to the experimental conditions (Fig. 1).

The experiment consisted of measuring the following electrophysiological parameters:

- Transepithelial potential difference (PD, mV)—changes in the transepithelial electrical potential measured under stationary conditions,
- Minimal and maximal transepithelial electrical potential difference (PDmin, PDmax, mV) measured during a 15-s stimulation,
- Transepithelial electrical resistance (R, Ω/cm^2^).

The experiment lasted 75 min for every tissue sample, including 30 min of the incubation and 45 min of the measurement. PD was recorded continuously, whereas R was determined by the acting on the tissue specimen with stimulus intensity current of ± 10 μA. Then, the corresponding voltage change was measured and the resistance was counted according to the Ohm's law.

### Data analysis

Data were recorded in the experimental protocol EVC 4000 (WPI, USA). It was connected to the data acquisition system MP150 and transferred to the computer data acquisition software AcqKnowledge 3.8.1 (Biopac Systems, Inc., USA).

The statistical analysis was made in the Statistica 11.00 software (StatSoft, Inc.). The data did not follow a normal distribution, which was verified by the Kolmogorov–Smirnov test with the Lilliefors correction. The Wilcoxon test was used to compare data from the same incubation conditions with a significant level *p* < 0.05. The Mann–Whitney test was used to detect significant differences (*p* < 0.05) for the different experimental conditions in the three types of tissue examined.

### Ethical approval

No experiments involving human participants were performed in the study. The present experiment did not include living animals and according to the Polish and European Union law, the bioethical committee agreement was not required. Animal care was in accordance with the guidelines and regulations as stipulated by the Polish Animal Protection Act and the European Directive on the Protection of Animals Used for Scientific Purposes (2010/63/EU). All applicable institutional and national guidelines for the care and use of animals were followed. The animals were housed in disposable cages and allocated 2 rabbits per cage, next were scarified in accordance with the guidelines and regulations of Nicolaus Copernicus University and by qualified personnel with all certificates for killing animals in the laboratory (no. 14/2016 from 10/20/2016), required by the UE and Polish law.

## Supplementary Information

Supplementary Information.

## Abbreviations

B: Bumetanide solution (0.1 mM)
CFTR: Cystic fibrosis transmembrane conductance regulator
ENaC: Epithelial sodium channel
Ni: Nickel chloride (0.1 mM) solution
PD: Transepithelial potential difference (mV)
PDmax: Maximal transepithelial potential difference (mV)
PDmin: Minimal transepithelial potential difference (mV)
R: Transepithelial resistance (Ω/cm^2^)
