# Supplementary material for: Nickel ions influence the transepithelial sodium transport in the trachea, intestine and skin
Source: Sci Rep. 2023 Apr 28;13:6931. doi: 10.1038/s41598-023-33690-2 (PMC10147918; doi:10.1038/s41598-023-33690-2)
Supplement: Supplementary file 1 — Supplementary Information. [file 41598_2023_33690_MOESM1_ESM.docx]

**Theme:** Nickel ions influence the transepithelial sodium transport in the trachea, intestine and skin.

Iga Hołyńska-Iwan^1*^, Marta Sobiesiak^2^, Wojciech Kowalczyk^3^, Marcin Wróblewski^4^, Anna Cwynar^1^, Karolina Szewczyk-Golec^4^

^1^ Department of Pathobiochemistry and Clinical Chemistry, Faculty of Pharmacy, Ludwik Rydygier Collegium Medicum in Bydgoszcz, Nicolaus Copernicus University in Torun, Poland;

^2^ Department of Inorganic and Analytical Chemistry, Faculty of Pharmacy, Ludwik Rydygier Collegium Medicum in Bydgoszcz, Nicolaus Copernicus University in Torun, Poland;

^3^ Clinic of Allergology, Clinical Immunology and Internal Diseases, Dr Jan Biziel's University Hospital No. 2, Bydgoszcz, Poland**;**

^4^ Department of Medical Biology and Biochemistry, Faculty of Medicine, Ludwik Rydygier Collegium Medicum in Bydgoszcz, Nicolaus Copernicus University in Torun, Poland;

**Short title**: Nickel influences sodium pathways.

Corresponding author:

Iga Hołyńska-Iwan

Laboratory of Electrophysiology of Epithelial Tissue and Skin,

Department of Pathobiochemistry and Clinical Chemistry,

Collegium Medicum in Bydgoszcz Nicolaus Copernicus University in Torun,

address: M. Skłodowskiej-Curie 9

85 – 094 Bydgoszcz, Poland

phone: (48) 525853598

email: [igaholynska@cm.umk.pl](mailto:igaholynska@cm.umk.pl)

ORCID: 0000-0002-0986-5604

Table A1. Results of the Wilcoxon test (*p*<0.05) for transepithelial electric potential measured during stationary conditions and during 15-sec stimulations for the control specimens.

| **Parameter** | ***p*** |
| --- | --- |
| **Trachea** | |
| PD/PDmax | ***0.000499*** |
| PD/PDmin | ***0.001773*** |
| PDmax/PDmin | ***0*** |
| **Colon** | |
| PD/PDmax | ***0*** |
| PD/PDmin | ***0.000043*** |
| PDmax/PDmin | ***0*** |
| **Skin** | |
| PD/PDmax | ***0*** |
| PD/PDmin | ***0*** |
| PDmax/PDmin | ***0*** |

Abbreviations: PD - transepithelial potential difference of tissue surface (mV) measured in stationary conditions, PDmin - minimal transepithelial potential difference measured during a 15-sec stimulation of the tissue surface (mV), PDmax - maximal transepithelial potential difference measured during a 15-sec stimulation of the tissue surface (mV).

Table A2. Results of the Wilcoxon test (*p*<0.05) for transepithelial electric potential measured during stationary conditions (PD) and during 15-sec stimulations for the specimens treated by the solution of nickel (Ni, 0.1 mM) and bumetanide (B, 0.1 mM).

|  | Stimulation | |
| --- | --- | --- |
|  | Ni | B |
| **Trachea** | *p* value | *p* value |
| PD/PDmax | ***0.000001*** | ***0*** |
| PD/PDmin | ***0*** | ***0*** |
| PDmax/PDmin | ***0*** | ***0*** |
| **Colon** | | |
| PD/PDmax | ***0*** | ***0*** |
| PD/PDmin | ***0*** | ***0*** |
| PDmax/PDmin | ***0*** | ***0*** |
| **Skin** | | |
| PD/PDmax | ***0*** | ***0*** |
| PD/PDmin | ***0*** | ***0*** |
| PDmax/PDmin | ***0*** | ***0*** |

Abbreviations: B – the bumetanide (0.1 mM) solution, Ni – the nickel chloride (0.1 mM) and B (0.1 mM) solution, PD - transepithelial potential difference of the tissue surface (mV) measured in stationary conditions, PDmin - minimal transepithelial potential difference measured during a 15-sec stimulation of the tissue surface (mV), PDmax - maximal transepithelial potential difference measured during a 15-swc stimulation of the tissue surface (mV).


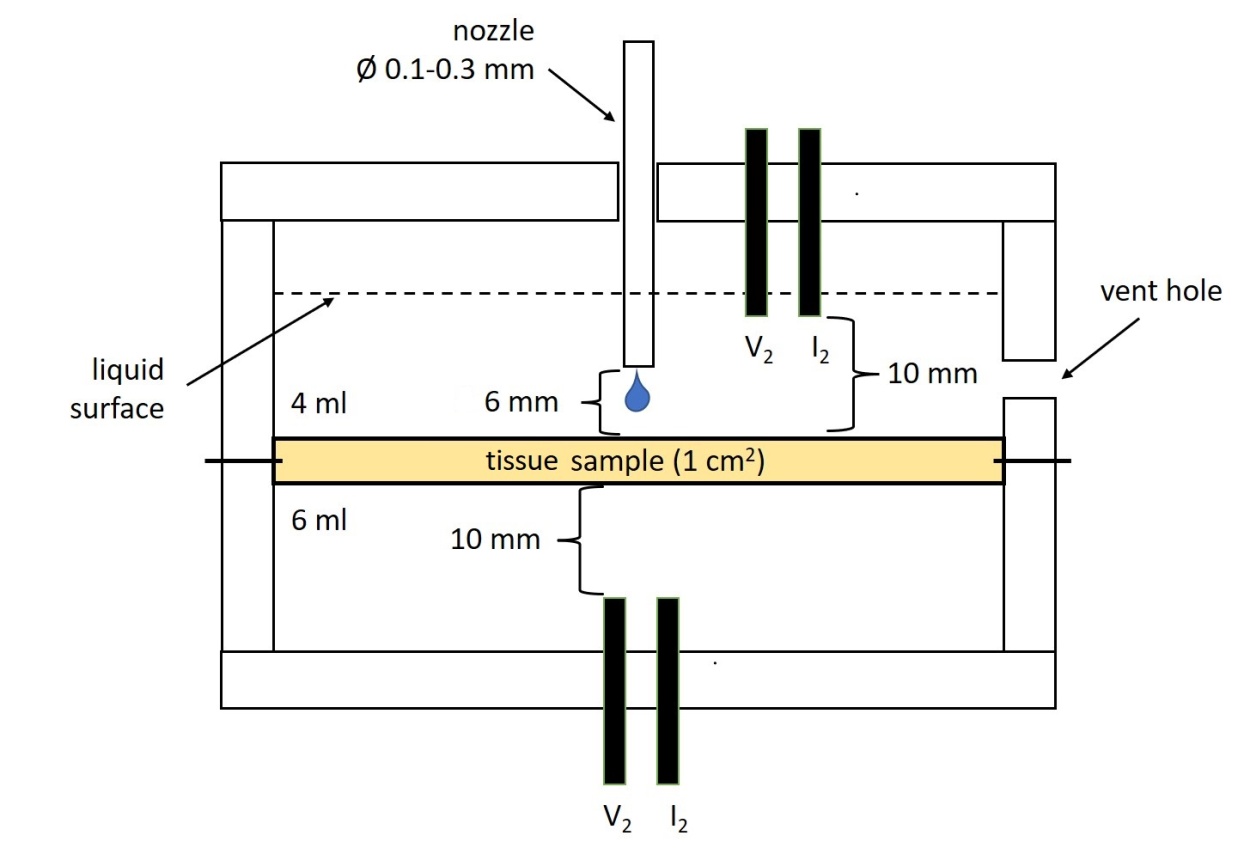


Figure A1. The scheme of modified Ussing chember.

Abbreviations: V1 - measured electrode, V2 - ground electrode, I1, I2 - electrode to pass the current of constant intenisty of 10 μA from I1 side and 10 μA from I2 side.

Nozzle - diameter 0.1 - 0.3 mm, put in the distance 10 mm from the tissue, is used for stimulation, the stimulation fluid with the volume 1.00 ml/15 s (0.06ml/s), according to experimental procedure.
